# Ethical considerations for biobanks serving underrepresented populations

**DOI:** 10.1111/bioe.13381

**Published:** 2024-12-10

**Authors:** Yoon Seo Lee, Nelson Luis Badia Garrido, George Lord, Zane Allan Maggio, Bohdan B. Khomtchouk

**Affiliations:** 1 Harvard John A. Paulson School of Engineering and Applied Sciences, Harvard University, Cambridge, Massachusetts, USA; 2 The College of the University of Chicago, Chicago, Illinois, USA; 3 Department of Biomedical Engineering & Informatics, Luddy School of Informatics, Computing, and Engineering, Indiana University, Indianapolis, Indiana, USA; 4 Krannert Cardiovascular Research Center, Indiana University School of Medicine, Indianapolis, Indiana, USA; 5 Center for Diabetes & Metabolic Disorders, Indiana University School of Medicine, Indianapolis, Indiana, USA; 6 Center for Computational Biology & Bioinformatics, Indiana University School of Medicine, Indianapolis, Indiana, USA; 7 Department of Medical and Molecular Genetics, Indiana University School of Medicine, Indianapolis, Indiana, USA

**Keywords:** Biobank, data sharing, health disparities, informed consent, precision medicine, sustainability

## Abstract

Biobanks are essential biological database resources for the scientific community, enabling research on the molecular, cellular, and genetic basis of human disease. They are crucial for computational, data‐driven biomedical research, which advances precision medicine and the development of targeted therapies. However, biobanks often lack racial and ethnic diversity, with many data sets predominantly comprising individuals of white, primarily northern European, ancestry. Establishing or enhancing biobanks for the inclusion of historically underrepresented populations requires meticulous ethical and social planning beyond logistical, legal, and economic considerations. This guide provides a roadmap for building and sustaining diverse biobanks, emphasizing ethical guidelines and cultural sensitivity. We highlight the importance of obtaining informed consent from donors, respecting their bodily autonomy, and the economic and research benefits of diverse biobanks to enable precision medicine, drug discovery, and industry‐academic partnerships. Prioritizing key ethical and social considerations allows biobanks to advance scientific knowledge while upholding the rights and autonomy of underrepresented populations. Diversity in biobank sample collection enhances research outcomes by ensuring findings are representative and applicable to various human population groups, fostering trust, promoting inclusivity, and addressing health disparities while informing health policy. This is vital to ensuring biobanking efforts contribute meaningfully to the advancement of health equity.

## 1 | INTRODUCTION

Biobanks are an integral tool in the field of precision medicine, as they support modern biomedical research through the collection, storage, and distribution of high‐quality biological samples and their associated data. While not all precision therapies are solely built upon or justified by genomics and tissue analysis, many in the field recognize the value that biobanks bring to enhancing our understanding and tailoring treatments to specific patient subpopulations. As the demand for personalized medicine, genomics, and biomarker discovery continues to grow, so does the need for robust, well‐managed, and diverse biobanks.

Diversity is crucial in personalized medicine because it ensures that treatments and medical interventions are effective across different populations. Diverse genetic data allows researchers to identify unique biomarkers and other pathophysiological insights, which are essential for developing tailored therapies. Biobanks play a pivotal role in this by collecting and storing biological samples from a wide range of individuals. This diversity in biobanks has already led to significant biomarker discoveries, such as those highlighted in the Pharma Proteomics Project.^1^ This biopharmaceutical consortium characterized the plasma proteomic profiles of 54,219 UK Biobank participants. The extensive and varied data provided by the UK Biobank enabled researchers to identify 14,287 primary genetic associations, with 81% being previously undescribed, and conduct ancestry‐specific protein quantitative trait locus (pQTL) mapping in non‐European individuals, who are typically underrepresented in these types of studies. The use of biobanks made this possible by offering a large, diverse data set that reflects the genetic variation within different populations, leading to more comprehensive and inclusive insights. The impact of such studies going forward includes improved drug discovery, better understanding of disease mechanisms, and the development of predictive models and therapeutics that are effective for a broader range of individuals, ultimately promoting health equity in personalized medicine.

However, many minority populations are still inadequately included in current biobanks, which underscores the need for expanding biobanks in these understudied communities to address existing genomic health disparities. Increasing representation of minority populations in biobanks can lead to innovative findings that have greater explanatory power in these populations relative to studies using less diverse data sets. For instance, Sohail et al. were able to make better predictions for 22 complex traits for Mexican populations using the data from the recently established Mexico Biobank Project (MXB) when compared to utilizing data from the UK Biobank.^2^ Building such biobanks for historically underrepresented populations of diverse ancestral backgrounds, or updating existing biobanks in order to prioritize their inclusivity, can help address a critical gap in achieving equitable healthcare outcomes. Underserved minority communities may currently lack access to large‐scale data, but initiatives to increase representation in biobanks can help achieve sufficient statistical power for research, advancing the goal of equitable and effective medicine for all.

However, establishing a biobank is a complex and multifaceted task that requires a team to be well‐prepared to address critical topics, such as cultural sensitivity and respect, to be successful. It requires not only an understanding of the scientific and technical aspects but also a deep appreciation of the ethical, legal, and social implications when working with minority populations of diverse ancestral backgrounds.

In this guide, based on our in‐house biobank and database expertise, we discuss some of the key ethical and social considerations in biobank design and management for historically underrepresented populations in the United States, further supplemented by our discussions with researchers and administrators at national biobanks, such as the Kaiser Permanente Research Bank and the Indiana Biobank at Indiana University.^3^ Our goal is to offer a valuable educational resource for individuals and institutions that are building a biobank for the first time, as well as for those who are looking to improve existing biobank operations.

This work addresses how to navigate the challenges and complexities associated with biobank development for underserved communities in the United States by covering topics ranging from ethical considerations and potential misuse of biological data, to community engagement and education. Given the diverse legal, cultural, and historical contexts in different nation‐states, we cannot provide a comprehensive guide that could address all different possible international scenarios, but the general principles outlined herein can be adapted to biobanks serving different communities within the United States of America.

## 2 | ETHICAL CONSIDERATIONS: INFORMED CONSENT AND DONOR AUTONOMY

Biobanks play a crucial role in advancing biomedical research by providing valuable human biospecimens and associated data. However, the collection, storage, and sharing of these resources raise several ethical concerns that need to be carefully addressed, especially regarding how to obtain informed consent, protecting participant privacy, ensuring cultural sensitivity and respect, and addressing other ethical challenges.^4^ Additionally, biobanks are considered to be legal entities according to the International Organization for Standardization,^5^ therefore creating a legal need, as well as a moral one, to properly handle and manage PII and PHI.

In particular, the issues surrounding informed consent, such as obtaining reconsent and privacy of personal data, have been an ongoing challenge with no clear‐cut resolution. As society evolves and new technological advancements emerge, especially with the rapid advancements being made with artificial intelligence (AI)‐assisted methods (more on this in the Case Studies section), the complexities surrounding informed consent are likely to persist. Researchers and ethicists continue to grapple with finding a balance between respecting donor autonomy and accommodating the dynamic nature of biomedical research.^6^ The need for flexible and adaptive consent models that can address these evolving challenges is necessary. Thus, informed consent will likely remain a critical and evolving issue, requiring continuous dialogue, innovation, and adaptation to ensure ethical standards are upheld and public trust is maintained in biobanking practices.

### 2.1 | Obtaining informed consent

Informed consent is a cornerstone of ethical practice in biobanking, safeguarding the autonomy and rights of all research participants. Its essence is not just in its acquisition but in its careful formulation and ongoing reaffirmation. Biobanks must provide comprehensive information about their objectives, the nature of samples and data collected, prospective use‐cases, and the inherent risks and benefits of participation, ensuring that consent documents comply with legal requirements across all operational locales.

However, with evolving objectives and potential uses, and the rapid rate of technological and biomedical research developments, the fluid nature of biobanking requires an adaptive approach to informed consent. Given that the full breadth of a biobank’s future operations might not be evident during the initial consent phase, it is advocated to envision the biobank as a “living” entity. This perspective promotes ongoing engagement with the community, especially members of the populations of interest, to monitor and provide input on biobank access and activities throughout its existence.

The scope of informed consent is vital and should be made clear to all prospective donors. Donors should understand that their consent covers both the physical biospecimens (urine, blood, saliva, etc.) and the data generated from them (Supporting Information S1: Table 1). However, while they have the right to request sample destruction at any time, this right may have limitations with regard to data previously shared or integrated into research practice. Once a retraction is received, any future utilization of already derived data should only proceed after engaging with donors, legally authorized representatives, or advocates of the population of interest. Proactive engagement is crucial when new applications for samples or data emerge, emphasizing community decision‐making in the dissemination of any findings.

The complexities surrounding informed consent have led to the rise of dynamic and tiered consent models. These models, instead of offering blanket permissions, give donors tailored choices about the use of their samples. Dynamic consent permits ongoing adjustments to permissions as research or personal preferences evolve, necessitating updated consent when introducing new developments. Conversely, tiered consent allows donors to set specific boundaries on the access or research types involving their data and samples, emphasizing donor control. Both models emphasize donor autonomy and enhance both transparency and trust in biobanking. Combining these approaches would likely be ideal for most applications, as it would respect donor autonomy while allowing for updated consent to be obtained in response to legal requirements and emerging technological and societal developments.

In addressing consent within biobanks, one should acknowledge the extensive work and initiatives that have set precedents to shape current practices. Initiatives such as the UK Biobank, NIH *All of Us* Research Program, BioVU (Vanderbilt University’s De‐Identified Biobanking Program), and the original Human Genome Project (HGP) Ethical, Legal, and Social Implications (ELSI) projects have all been instrumental in shaping the landscape of consent in biobanking. The UK Biobank, with its large‐scale biomedical database and research resource, provides a robust framework for informed consent, emphasizing transparency and participant engagement. Similarly, NIH biorepositories such as *All of Us* or BioLINCC, which focus on the diverse representation of participants, have implemented innovative tiered approaches to consent, ensuring inclusivity and respect for individual autonomy at various comfort levels. BioVU, as a DNA biobank linked with electronic medical health records, highlights the importance of dynamic consent in the context of continually evolving data, while the HGP ELSI programs have laid the groundwork for ethical considerations in genetic research, including consent practices. These initiatives highlight the evolution of consent practices, underscoring the importance of participant‐centric approaches and the continuous refinement of consent processes in biobanks.

By prioritizing transparency, respect, and the comfort of donors, biobanks can solidify the principles of autonomy and donor agency, therefore fostering trust throughout the entire research journey. This is particularly crucial for building and maintaining biobanks focused on historically underrepresented populations, as one must address the unique challenges and opportunities in working with these communities to ultimately reduce genomic health disparities. Approaching these challenges with cultural sensitivity and a commitment to non‐exploitative practices ensures that the research process honors the values and concerns of every community. This can be achieved by actively involving community leaders and stakeholders in decision‐making, so biobanks can promote transparent ethical standards and equitable benefits, thus strengthening the trust and participation of these vital groups.

### 2.2 | Importance of donor autonomy

Maintaining respect for donor autonomy necessitates acknowledging and honoring the right to withdraw consent at any time. During the initial consent process, it is necessary to include statements that stress voluntary participation and the possibility of withdrawal at any stage, as trust and transparency are critical to successful engagement.

However, the practicality of revoking consent can present certain limitations, so anticipating and planning for such events is necessary. For example, in the case of organ or cadaver donations, complications can arise when an individual may consent to a donation, but post‐mortem, family members might not concur with the deceased’s wishes. Family disputes might also emerge, where one member wishes to honor the deceased’s consent, but others may dissent. In all cases, the policy should be to respect the decision to withdraw consent, regardless of when it is made, if possible. On the other hand, jurisdictions may have established protections in place that make this matter even more complex. For example, the Uniform Anatomical Gift Act can prevent the next‐of‐kin from legally over-ruling the deceased’s wishes.^7^ It is a difficult situation that may arise, and so biobanks should have a process in place for such scenarios. For example, biobanks may consider immediately withdrawing consent to avoid causing further distress to families.

Additionally, pre‐mortem registered donors may change their mind later in life. In the case of living donors, occasional requests to cease participation in the study do arise. Commonly, these requests come from individuals who were initially comfortable participating when they were healthy, but later express concerns as they perceive a decline in their performance on health tests. These concerns may stem from new diagnoses or simply their reactions to being further tested. In these cases, all the individual’s files must be destroyed, save for the record of their initial registration and subsequent consent withdrawal, though it is important to note that some pre‐mortem research protocols perform research on data collected before death, and withdrawal options vary, with some allowing for data to be retained up to the point of withdrawal.

For longitudinal studies, it is not uncommon to have a participant’s family member or caregiver initiate the withdrawal, particularly when they believe they are safeguarding an individual who lacks the cognitive ability to make the decision themselves. It may be possible to encounter situations in which there is a clear conflict between the participant’s wishes and their next‐of‐kin. With thoughtful preparation and transparent communication with all individuals involved, biobanks can navigate such complex ethical dilemmas.

It is also important to be clear about how corporate entities and partners may profit from donor samples in the future. Offering compensation can compromise the ethical underpinnings of donation, potentially shifting the altruistic nature of their contribution. Donors are typically asked to view their samples as “gifts to science,” in order to help aid the progression of human health and knowledge. To enhance this trust, biobanks can offer donors the option to restrict their data from corporate access, as this choice may influence participation decisions. Expanding tiered consent to have more granular options for data sharing, limiting access of donor data to commercial entities, is one way of addressing this. Offering and respecting such preferences aligns biobanking with higher ethical standards. While this may limit a biobank’s appeal to researchers, ultimately, respecting the autonomy of donors takes precedence, and thus it is necessary to approach donation requests and patient recruitment with this in mind.

When building biobanks focused on historically underrepresented populations, it is essential to consider additional layers of autonomy and consent, especially when dealing with communities that may have heightened concerns due to past abuses or mistrust in medical research. Engaging community leaders and stakeholders in the consent process and ensuring clear, culturally sensitive ongoing communication can help in preparing for and addressing these challenges to foster a more inclusive biobank infrastructure.

### 2.3 | Protecting participant privacy

Participant privacy is a major ethical concern in biobanking, as the collected biospecimens and data often contain sensitive personal information. Even when collected information has been de‐identified, it is increasingly possible to re‐identify individuals based on their health data, especially given potentially geolocatable markers and poor privacy practices on the internet.^8^ This is a major concern for individuals from underrepresented communities because these communities are often smaller, making it easier to identify individuals within the group. To address this concern, biobanks are required to adhere to rigorous privacy protection measures, such as the Health Insurance Portability and Accountability Act (HIPAA) and Human Subjects Research Regulations (HSRR) laws, as overseen by an Institutional Review Board (IRB). The IRB is tasked with ensuring compliance with the Common Rule when research involves collecting protected health information (PHI) or personally identifiable information (PII). Thus, the collection of biological data and its associated descriptive metadata necessitates approval from the IRB.^9^

Despite the existence of fairly stringent privacy laws, it is also common for loopholes to be found within these laws and so, as a consequence, health information may be at risk of misuse. As the American Heart Association (AHA) states in their policy statement from August 2023: “…although both HIPAA and HSRR generally require explicit informed consent from a patient or research participant for the collection, sharing, and use of health information, they are limited to only individually identifiable information and cover only certain kinds of interactions, and there are many exceptions for minimal‐risk research that may be done in many cases without the contributors’ consent or knowledge…[and] virtually no regulations comprehensively cover the collection, sharing, and use of health and health‐proxy information by commercial companies.”^10^ While it is necessary for biobanks to follow the rules and regulations in place, it is recommended that these be further expanded upon in order to best protect the privacy of the subjects whose data is analyzed and shared as part of collaborative and commercial activities.

Through the emphasis on participant privacy and collaboration with IRBs, and any privacy officers separate from them, biobanks can maintain public trust, uphold legal obligations, and adhere to ethical regulations surrounding the use of sensitive personal information. The implementation of enhanced privacy measures and transparent communication is critical in protecting individuals from historically underrepresented populations who may have greater sensitivities regarding data use and gaining their confidence and participation.

## 3 | ETHICAL CONSIDERATIONS: RESPECTING DIVERSE BELIEFS AND VALUES

### 3.1 | Ensuring Cultural Sensitivity and Respect

Biobanks must recognize and respect the diverse cultural beliefs and values of the communities they serve. This involves engaging with community members, understanding their perspectives, and addressing their concerns regarding biobanking practices. Ensuring cultural sensitivity and respect also includes incorporating deep community input into the development of biobank policies, particularly regarding the collection, storage, and sharing of biospecimens and data. By fostering a culture of respect and understanding, biobanks can promote inclusivity and ensure the equitable distribution of research benefits, and recognizing specific cultural beliefs that may influence participation in biobanking is crucial for ethical practice. Previous studies on understanding the differences in values, norms, and expectations from various communities and cultures have shown that culturally grounded strategies are needed for community engagement, which could help increase minority population participation in future research.^11^

For example, the Native Hawaiian concept of “mana” exemplifies a cultural belief that can pose challenges to biobanking practices. In Native Hawaiian culture, mana is a fundamental and intricate belief system. It is often described as a supernatural force or spiritual power residing in people, animals, plants, and even inanimate objects and is integral to one’s identity. This concept is profoundly connected to various body parts and elements. Native Hawaiians believe that “[personal] possessions, clothing, body parts and even exuviae of the body [are] considered to be inextricably tied to the individual.” The belief that these items contain an individual’s *mana* means they must be treated with great respect and care.^12^ This deeply‐held belief in the spiritual and life forces contained within the body and its parts can make members of the Native Hawaiian community hesitant to participate in tissue donations for biobanking. Therefore, the process of obtaining consent and any subsequent donations must be approached with an understanding and respect for this belief. Cultural sensitivity is essential, making it crucial to involve community members from the population of interest in the development of ethical biobanking practices.

Engaging community leaders and representative organizations from minority communities is highly encouraged, and even necessary, to do right by these communities. Historically, many minority communities have experienced unethical research practices, leading to a deep mistrust of scientific research. For instance, the infamous Tuskegee Syphilis Study and the exploitation of Henrietta Lacks’ cells without her consent have left indelible marks on the collective memory of many African American communities. As a result, there is a strong sense of reservation and feelings of exploitation among these groups when it comes to participating in research. To address these concerns, biobanks must engage in meaningful and sustained dialogue with community leaders and representative organizations. This engagement should not be superficial but rather a committed partnership where community voices are heard and valued. The following recommendations should be kept in mind for successful engagement: (1) build and foster relationships with community leaders to build trust and collaborate with during research outreach, (2) train biobank staff on cultural sensitivity to ensure historical and cultural context remain at the forefront of interactions and decision‐making, (3) establish dedicated community advisory boards to receive feedback on ethical issues, appropriate cultural practices, and areas of importance for the members of said communities, (4) ensure community members are well‐informed on how their biospecimens and data are being used, with an emphasis on progress made in research stemming from the use of this data and providing answers to any questions and concerns raised, (5) prepare a thorough and culturally‐sensitive informed consent process that uses transparent language and helps any layperson understand the decisions being presented to them, (6) create accessible methods with which community members can reach researchers and provide feedback or raise concerns regarding the use of their data ‐‐ this feedback should be taken seriously and addressed appropriately when possible, (7) communicate the potential benefits of the research, including updates in language that is accessible, especially those on advancements in healthcare, and offer opportunities for qualified and interested community members to learn about the research methods and findings.

### 3.2 | Communicating the benefits of biobanking

Biobank sustainability is challenged by the insufficient data available to describe the outputs and benefits produced by biobanking.^13^ Thus, effectively communicating the benefits of biobanking to the public, research communities, and other stakeholders can help garner support and participation. This involves highlighting the value of biomedical research and making its purpose more easily understood for the layperson, but especially highlighting the value of biobanks in accelerating such research, and ultimately contributing to the development of new diagnostics, treatments, and preventive strategies for all interested parties. An example of such an initiative is the Ola HAWAII specialized center, which received funding renewal from the National Institute on Minority Health and Health Disparities from 2022 to 2027, via grant #2U54MD007601‐36. The center has the aim of “improving minority health and reducing disparities for those communities in Hawaiʻi which suffer disproportionately in health outcomes and healthcare access.”^14^ Part of the success of this initiative can be attributed to its regular reports back to the communities it serves on planned, ongoing, and completed research. By promoting a clear understanding of biobanking’s benefits, communities can become better informed and motivated to participate in or support biobanking initiatives.

### 3.3 | Examples of misuse of biological data obtained from minority populations

A glaring illustration of the ethical pitfalls tied to population data misuse is the lawsuit involving Bristol‐Myers Squibb and Sanofi against the State of Hawai’i. The two companies faced accusations of breaching Hawai’i’s Unfair and Deceptive Acts or Practices law, alleged to have misled the public about their antiplatelet drug, Plavix. Despite their awareness in 1998 of the drug’s diminished effectiveness for populations of Asian descent, a warning was only appended to the drug’s packaging 12 years later.^15^

Another example of the misuse of genetic data is that of the Havasupai Tribe in Arizona, USA. In the early 1990s, researchers from Arizona State University collected blood samples from Havasupai Tribe members, ostensibly to study the high prevalence of diabetes within the tribe. However, the samples were later used for other research purposes, including studies on schizophrenia and population genetics, without the explicit consent of the individuals or the tribe. The population genetics research, in particular, led to conclusions about the tribe’s geographical origins that contradicted their traditional beliefs and oral histories, causing significant distress and feelings of violation within the community.^16^ Therefore, data acquisition and application related to specific populations must be approached with utmost consideration. Misuse, especially when it jeopardizes the well‐being of individuals within these communities, must be stringently avoided.

However, navigating these circumstances can become complex, especially when attempting to generalize to larger populations, as reflected in the challenges surrounding data use ethics and consent protocols. A study that surveyed Native Hawaiians reported that 78% of the participants would prefer to be re‐consulted for consent when re-using identified specimens, with approximately 35% expressing the same sentiment for anonymized specimens.^17^ Although these findings cannot be generalized to the entire Native Hawaiian population, they underscore the importance of upholding individual autonomy and rights over personal biological data, more so as biobanks endeavor to incorporate diverse and historically underrepresented populations.

Arguments have been made to say that research on individuals within a population subjects the entire group to shared adverse consequences that may result from involvement in research.^18^ This highlights the urgent need to ensure that data obtained from these underrepresented communities is not only used for driving scientific progress but also for serving the interests of the communities from which it originates. Misuse of biological data, particularly from these communities, not only violates ethical principles but also risks further mistrust.

Going forward, biobanks and the larger research community must diligently strive to alleviate such ethical dilemmas. Potential strategies to achieve this could involve improving informed consent procedures, such as the notion of seeing the biobank as a “living” entity discussed previously, developing policies for equitable sharing of research benefits, such as community ownership and dissemination of findings, and forming respectful partnerships with populations of interest to ensure that any and all tailored ethical guidelines are followed. By using such collective efforts, biobanks can fuel biomedical research while upholding the rights and welfare of the individuals whose data they house.

## 4 | CASE STUDIES OF BIOBANKS IN MODERN BIOMEDICAL RESEARCH

Biobanks are revolutionizing biomedical research by advancing precision medicine, enhancing drug discovery, and fostering collaborations between academic institutions and industry. Significant financial investments and commercial potential associated with biobanks help highlight their role in enabling innovative therapies, facilitating AI‐driven research, and promoting the development of personalized treatments. Various case studies highlight the increasing importance of biobanks in the clinic and their influence on academic research and the biotechnology industry. In recent years, there has been an emergence of a new trend wherein an increasing number of companies are leveraging AI and machine learning to enhance traditional drug discovery methods. These novel approaches, often characterized as “no‐hypothesis” target discovery, can mitigate the inherent risks associated with the conventional drug development process, which relies heavily on preclinical studies to identify and validate prospective drug targets and mechanisms. An example of this is the work of BioAge Labs, which used AI to analyze clinical and omics data from a human aging study cohort, thereby identifying a novel drug for muscle atrophy, BGE‐105.^19^ Following administration of this drug, a highly selective agonist of the apelin receptor APJ, BioAge Labs observed a statistically significant effect in preventing muscle atrophy compared to a placebo in healthy volunteers aged 65 or older after ten days of bed rest.^20^ This success story, underpinned by data stored in and retrieved from biobanks, reaffirms the crucial and continuing role of these resources in future biomedical research.

Another example, Prometheus Biosciences (acquired by Merck for US $10.8 billion) leveraged precision medicine to devise innovative drugs and diagnostic tools for inflammatory bowel disease (IBD).^21^ The company criticized current IBD treatments as being overly generalized and failing to consider biological diversity. In order to create more specialized IBD treatments, Prometheus unveiled Prometheus360, a technology platform that includes a biological database and biobank of specimens from patients suffering from IBD and other gastrointestinal disorders. Named the MIRIAD Biobank (Mucosal Immunology Repository for Inflammatory and Digestive Diseases), it holds over 20,000 samples amassed over two decades and was exclusively licensed from the University of California Los Angeles (UCLA) Cedars‐Sinai Medical Center.^22^ Prometheus, in its initial public offering (IPO) prospectus, asserted that the application of machine learning analysis to these longitudinal clinical samples, along with detailed genotyping information on IBD loci, fosters the development of precision therapies and accompanying diagnostics. The company’s successful IPO, which raised over US $190 million for further research,^23^ demonstrates the financial and academic potential of long‐term data collection via biobanks.

Similarly, in 2020, Bristol Myers Squibb (BMS) purchased MyoKardia for approximately US $13.1 billion.^24^ A prime motivation for this acquisition was the drug mavacamten, a potential first‐in‐class cardiovascular medicine for obstructive hypertrophic cardiomyopathy (HCM), whose development was made possible due to MyoKardia’s collaboration with SHaRe, an international repository of clinical data^25^ that is integral to their precision medicine platform. Another motivation for this acquisition was MyoKardia’s internal biobank on over 16,000 cardiomyopathy patients with proprietary whole‐exome sequencing data, which was used to not only identify the company’s lead drug candidate for the treatment of HCM (MYK‐461, which later became mavacamten), but also to kickstart new phase 1 clinical trials in dilated cardiomyopathy (DCM) based on novel druggable targets identified in the biobank. MyoKardia’s use of its precision medicine platform, which combines disease research, drug discovery, and clinical science in a reciprocal cycle, facilitated the continuous identification of new disease targets and potential clinical treatments. In 2016, mavacamten received the Orphan Drug Designation by the FDA.^26^

Continuing the trend of acquisitions of biobanks by large pharmaceutical companies, in 2012, Amgen announced its acquisition of deCODE Genetics, a biopharmaceutical genetics research company based out of Iceland. With a $401 million cash transaction, Amgen gained access to deCODE’s extensive Icelandic biobank that genotyped over two‐thirds of the adult population, which enabled the identification of genetic risk factors across various diseases from cardiovascular conditions to cancer.^27^ The merger aimed to combine deCODE’s genetic research with Amgen’s drug development initiatives—a strategy expected to expedite the discovery, validation, and prioritization of novel therapeutic targets.^28^ A prime example that resulted from this merger is AMG 133, a novel bispecific glucose‐dependent insulinotropic polypeptide receptor (GIPR) antagonist and glucagon‐like peptide‐1 (GLP‐1) receptor agonist molecule. Eventually, Amgen moved forward with phase 1 clinical trials, in which subcutaneous injection of AMG‐133 was associated with a 14.5% reduction in body weight after 12 weeks at the highest dose tested.^29^ This union underscores the significant potential of large‐scale biobanks in accelerating the delivery of impactful therapies to the market.

In 2017, an analysis was conducted on Auria Biobank, Finland’s foremost clinical biobank containing over a million samples, closely associated with Turku University in partnership with the FinnGen consortium.^30^ This analysis examined the biobank’s efforts to develop business operations centered around its collection of tissue samples and patient records. Auria Biobank aims to advance Finnish biomedical research through the public dissemination of summary‐level statistics data and controlled‐access dissemination of individual‐level genetics data sets to academic institutions, finding that commercialization and partnerships with major pharmaceutical companies, such as Novartis, Bayer, and Roche, significantly support this goal.^31^ These collaborations not only provide essential funding but also facilitate the translation of research into practical medical applications, highlighting the critical role that commercial partnerships play in the sustainability and innovation potential of biobanks.

Lastly, the collaboration between Rush University Medical Center (Rush) and Tempus Labs, a company focused on personalized cancer care, is an example of another successful academic‐industry partnership. Tempus, a technology company, is constructing the world’s largest molecular and clinical data library and a system to make this data accessible and useful.^32^ Rush, a major healthcare provider in Chicago, Illinois, has paired with Tempus to become a leading cancer treatment center. This collaboration has Tempus storing and analyzing Rush tissue samples, providing crucial findings to clinicians, and augmenting its growing cancer genetics and molecular function database.^33^ The collaboration has led to the development of 3D organoids and other in‐vitro and in‐vivo models, enabling Rush researchers to test potential patient responses to specific treatments—a crucial step toward more personalized cancer care. The insights and analyses from Tempus can enable Rush clinicians to better comprehend their patients’ statuses and diagnoses, thus equipping them with the best tools for treatment, offering cancer patients increased hope for remission and a healthy life. This partnership is a testament to how biobank and industry collaboration can advance biomedical research when done correctly.

There is clear evidence, both from a commercial and academic standpoint, that biobanks play a pivotal role in advancing biomedical research, as demonstrated by the case studies discussed above. Diversifying biobank data sets will not only enhance the statistical power of research but also improve the generalizability of findings, enabling these results to benefit a wider range of populations. The inclusion of underrepresented groups in biobanks helps ensure that the outcomes of biomedical research are more equitable and applicable to broader demographics, reducing health disparities and promoting precision medicine for all. As the NIH *All of Us* Research Program eloquently explains: “A crucial part of improving health outcomes for everyone means addressing health inequities, and addressing health inequities is impossible without a data set that includes individuals from backgrounds and life circumstances that put them at greater risk of experiencing those health inequities.”^34^

While commercial interest contributes significantly to biobank finances, this interest typically stems from the intrinsic value that large diverse data sets hold. The more comprehensive and heterogeneous a set of biospecimen samples stored and digitized into a biological database, the more robust and statistically significant the research outcomes, which is why both academic and commercial entities are drawn to large biobank collections. However, the pursuit of commercial success need not come at the expense of ethical considerations or the wishes of those who contribute their data. For example, the successful implementation of tiered and dynamic consent models allows participants to decide how their data is used, helping to ensure that those who wish to restrict the commercial use of their data are respected. These models provide flexibility, enabling contributors to optout of specific research avenues or commercial ventures while still allowing their data to be used in academic research or public health initiatives.

Biobanks can remain commercially viable while upholding the ethical principles of transparency and respect for participant autonomy. By honoring data exclusion requests, biobanks maintain the trust of their participants, which is essential for their long‐term sustainability and growth. Ultimately, biobanks can serve as a bridge between academic and commercial interests, fostering collaborations that accelerate drug discovery and the development of innovative therapies. Through the promotion of diverse and inclusive data sets, biobanks can enhance the quality and impact of biomedical research while ensuring that the benefits of these advancements are shared more broadly across different populations.

## 5 | EXPANDING POPULATIONS OF INTEREST WHILE ENSURING CULTURAL SENSITIVITY

The true value of a biobank lies in the depth and breadth of data it curates and can produce with evolving advancements. Ensuring diversity across racial, gender, religious, dietary, clinical, geographical, and other dimensions to encompass new populations of interest can enrich the biobank’s data profile. Such comprehensive data not only bolsters its role in biomedical research but also paves the way for the development of more tailored and holistic treatments. At a minimum, it helps prevent harm to specific populations, as illustrated by the previously discussed case involving the State of Hawai’i, Bristol‐Myers Squibb, and Sanofi.

Broadening the demographic scope of a biobank also presents a unique opportunity to democratize access to cutting‐edge research for marginalized and underrepresented communities. These communities, often left on the periphery of major research initiatives, can greatly benefit from research tailored to their specific genetic and health profiles.^35^ This is also exemplified by the work of Sohail et al. mentioned earlier. Beyond this, minority populations benefit from this demographic broadening simply from having more equitable increased representation in medical research, legislation, and planning as a whole. For example, a review of 32 commonly used federal health data systems conducted by the Association of State and Territorial Health Officials revealed that several U.S. overseas territories are presently underrepresented in these systems, with American Samoa missing from 84% of them, the Commonwealth of the Northern Mariana Islands from 75%, U.S. Virgin Islands from 69%, Guam from 59%, and Puerto Rico from 47%.^36^ Expanding representation in health systems and biobanks allows overlooked populations to receive better care and benefit more fully from biomedical research.

However, this expansion can introduce unique additional challenges, such as regional laws, cultural nuances, and educational disparities. Additionally, the logistical intricacies of sourcing samples from highly specific or niche populations cannot be underestimated. Yet, despite these hurdles, prioritizing inclusivity and equity in biobanks is crucial to ensure access to research for all communities, ultimately enhancing its value and enriching the scope of potential research collaborations. The best way to overcome these hurdles is by creating relationships that are built on clear and open communication, both with members of these communities and with those who represent them. Informed consent helps build trust, and this is necessary for patients to feel empowered to make their own decisions regarding their participation in research.

Moreover, long‐term engagement becomes possible when trust and respect are earned, leading to ongoing participation and collaboration, which can ultimately lead to richer, more representative data. This is also necessary for overcoming historical barriers to research by reducing fears of exploitation and unfair treatment, ensuring benefits from research are equally shared. When communities trust that their participation can help lead to safer and more effective treatments or, at the very least, help decrease negative outcomes by avoiding interventions not suitable for their unique genetic and physiological differences, they are more likely to take part in research and support initiatives that facilitate this. In this way, trust not only promotes participation but also enhances the quality and relevance of the research itself, ultimately benefiting the populations of interest in the long run.

One initiative aimed at increasing the representation of a specific population in biomedical research, in order to help address their unique health challenges, is the OurHealth Study. Founded by cardiologists and researchers at Massachusetts General Hospital and the Broad Institute of MIT and Harvard, this non‐profit initiative aims to uncover genetic factors influencing cardiovascular disease risk in South Asian populations, who have twice the risk compared to those of European ancestry. By collecting data from various ethnic groups of South Asian ancestry, the study seeks to address health disparities and improve treatments, highlighting the importance of diverse representation in research for broader applicability and effectiveness.^37^ Initiatives like this are crucial for improving the quality of the data analyzed and the generalizability, and thus the impact, of the findings generated from it.

## 6 | CONCLUSION

Biobanks play a crucial role in the progress of biomedical research by providing researchers with access to high‐quality biospecimens and data, which accelerates scientific discoveries and enables the development of novel diagnostics, therapeutics, and personalized medicine approaches. They significantly contribute to our understanding of the etiology and pathogenesis of various diseases, supporting the development of effective preventive strategies and improved patient outcomes. Moreover, biobanks are instrumental in biomarker discovery, identifying biological markers that can predict disease risk, progression, and response to treatment. Their use streamlines advancements in biomedical research, addresses longstanding gaps in healthcare, and leads to newer and more efficacious therapies.

However, building and sustaining a biobank for underrepresented populations can be full of ethical, legal, and social considerations. Implementing a robust ethical framework and actively engaging with these communities are essential to effectively address health disparities. Ensuring long‐term sustainability requires a proactive approach to overcoming challenges, adopting best practices, and maintaining transparency and accountability, particularly concerning data from minority and underrepresented groups. With an ongoing focus on improvement and transparency, a biobank can continue to evolve alongside scientific progress and retain the trust of both researchers and the communities it serves.

In conclusion, establishing a successful biobank is a complex endeavor, but with careful planning and execution, it can become an invaluable asset to the scientific community and promote equity in biomedical research. Numerous ethical challenges exist in the biobanking space, especially concerning data from underrepresented populations. However, by anticipating these challenges, preparing adequately, and incorporating input from these communities while respecting historical and cultural contexts, biobanks can successfully integrate data from these understudied, underserved, and underrepresented (U3) groups. By addressing the challenges and embracing the opportunities presented, a new biobank can support the ongoing advancement of biomedical research, ultimately leading to improved patient care and outcomes for all. The future of biobanking depends on the collective efforts of researchers, biobank managers, and policymakers to ensure that these critical resources continue to serve the diverse and evolving needs of the global scientific community.

## Supplementary Material

Supinfo

## References

[1] Sun BB, Chiou J, Traylor M, Benner C, Hsu YH, Richardson TG, Surendran P, Mahajan A, Robins C, Vasquez‐Grinnell SG, Hou L, Kvikstad EM, Burren OS, Davitte J, Ferber KL, Gillies CE, Hedman ÅK, Hu S, Lin T, … Whelan CD (2023). Plasma proteomic associations with genetics and health in the UK Biobank. Nature, 622(7982), 329–338. 37794186 10.1038/s41586-023-06592-6 PMC10567551

[2] Sohail M, Palma‐Martínez MJ, Chong AY, Quinto‐Cortés CD, Barberena‐Jonas C, Medina‐Muñoz SG, Ragsdale A, Delgado‐Sánchez G, Cruz‐Hervert LP, Ferreyra‐Reyes L, Ferreira‐Guerrero E, Mongua‐Rodríguez N, Canizales‐Quintero S, Jimenez‐Kaufmann A, Moreno‐Macías H, Aguilar‐Salinas CA, Auckland K, Cortés A, Acuña‐Alonzo V, … Moreno‐Estrada A (2023). Mexican Biobank advances population and medical genomics of diverse ancestries. Nature, 622(7984), 775–783. 37821706 10.1038/s41586-023-06560-0 PMC10600006

[3] Feigelson HS, Clarke CL, Van Den Eeden SK, Weinmann S, Burnett‐Hartman AN, Rowell S, Scott SG, White LL, Ter‐Minassian M, Honda SAA, Young DR, Kamineni A, Chinn T, Lituev A, Bauck A, & McGlynn EA (2022). The Kaiser Permanente Research Bank Cancer Cohort: A collaborative resource to improve cancer care and survivorship. BMC Cancer, 22(1), 209. 35216576 10.1186/s12885-022-09252-6 PMC8876075

[4] Caulfield T, & Murdoch B (2017). Genes, cells, and biobanks: Yes, there’s still a consent problem. PLoS Biol, 15(7), e2002654. 10.1371/journal.pbio.2002654 PMC5526496 28742850

[5] Annaratone L, De Palma G, Bonizzi G, Sapino A, Botti G, Berrino E, Mannelli C, Arcella P, Di Martino S, Steffan A, Daidone MG, Canzonieri V, Parodi B, Paradiso AV, Barberis M, & Marchiò C (2021). Basic principles of biobanking: From biological samples to precision medicine for patients. Virchows Arch, 479(2), 233–246. 34255145 10.1007/s00428-021-03151-0 PMC8275637

[6] Caulfield, & Murdoch, op. cit. note 4.

[7] Uniform Law Commission. (2009). Uniform Anatomical Gift Act. https://www.uniformlaws.org/viewdocument/final-act-19?CommunityKey=015e18ad-4806-4dff-b011-8e1ebc0d1d0f

[8] Price WN II (2017). Risk and resilience in health data infrastructure. Colorado Technology Law Journal, 16(1), 65–101.

[9] Grigorenko EL, & Bouregy S (2009). Biobanking on a small scale: Practical considerations of establishing a single‐researcher biobank. https://www.semanticscholar.org/paper/Biobanking-on-a-Small-Scale%3A-Practical-of-a-Biobank-Grigorenko-Bouregy/9e2938d8dea0ea4c8361cf2f5f851d7d23fe5c23

[10] Spector‐Bagdady K, Armoundas AA, Arnaout R, Hall JL, Yeager McSwain B, Knowles JW, Price WN 2nd, Rawat DB, Riegel B, Wang TY, Wiley K Jr., & Chung MK (2023). Principles for health information collection, sharing, and use: A policy statement from the American Heart Association. Circulation, 148(9), e000084. 10.1161/CIR.0000000000001173 PMC10912036 37646159

[11] Memon R, Asif M, Khoso AB, Tofique S, Kiran T, Chaudhry N, Husain N, & Edwards SJL (2021). Recognising values and engaging communities across cultures: Towards developing a cultural protocol for researchers. BMC Medical Ethics, 22(1), 47; 33902560 10.1186/s12910-021-00608-4 PMC8072318

[12] Crabbe K, Fox K, & Coleman H (2017). Mana Lāhui Kānaka. Office of Hawaiian Affairs.

[13] Rush A, Catchpoole DR, Ling R, Searles A, Watson PH, & Byrne JA (2020). Improving academic biobank value and sustainability through an outputs focus. Value Health, 23(8), 1072–1078. 32828220 10.1016/j.jval.2020.05.010

[14] Ola HAWAII. (n.d.). https://reporter.nih.gov/search/EXXnfSVz6kenPI_8pHMLCA/project-details/10556969

[15] Eddins J (2023). State v. Bristol‐Myers Squibb Company, No. SCAP‐21‐0000363. https://law.justia.com/cases/hawaii/supreme-court/2023/scap-21-0000363.html

[16] Sterling RL (2011). Genetic research among the Havasupai‐‐a cautionary tale. Virtual Mentor, 13(2), 113–117. 23121851 10.1001/virtualmentor.2011.13.2.hlaw1-1102

[17] Fong M, Braun KL, & Chang RM (2004). Native Hawaiian preferences for informed consent and disclosure of results from research using stored biological specimens. Pacific Health Dialog, 11(2), 154–159. 16281693

[18] Ibid.

[19] Eisenstein M (2022). Machine learning powers biobank‐driven drug discovery. Nature Biotechnology, 40(9), 1303–1305. 10.1038/s41587-022-01457-1 36050552

[20] Lifespan.io. (2022). BioAge announces positive results against muscle atrophy. https://www.lifespan.io/news/bioage-announces-positive-results-against-muscle-atrophy/

[21] Vinluan F (2021). Prometheus Bio’s IPO raises $190 M for inflammatory bowel disease R&D. MedCity News. https://medcitynews.com/2021/03/prometheus-bios-ipo-raises-190m-for-inflammatory-bowel-disease-rd/

[22] Cedars‐Sinai Medical Center & Precision IBD, Inc. (2017, September). Exclusive license agreement. https://www.sec.gov/Archives/edgar/data/1718852/000119312521049126/d14529dex1012.htm

[23] Prometheus Biosciences, Inc. (2023, April 15). Form 8‐K. CURRENT REPORT Pursuant to Section 13 or 15(d) of the Securities Exchange Act of 1934. https://www.sec.gov/Archives/edgar/data/1718852/000119312523102779/d462383ddefa14a.htm

[24] Bristol Myers Squibb. (2020, October 5). Bristol Myers Squibb to acquire MyoKardia for $13.1 billion in cash. https://www.sec.gov/Archives/edgar/data/14272/000114036120022458/brhc10015772_ex99-1.htm

[25] BioSpace. (2014, May 14). MyoKardia announces launch of SHARE patient registry for genetic heart disease in collaboration with world‐leading cardiovascular patient care centers. https://www.biospace.com/article/releases/myokardia-announces-launch-of-share-patient-registry-for-genetic-heart-disease-in-collaboration-with-world-leading-cardiovascular-patient-care-centers/

[26] Myokardia, Inc. (2016, September 16). Amendment no. 1 to form S‐1 registration statement under the Securities Act of 1933. https://www.sec.gov/Archives/edgar/data/1552451/000119312516719919/d260307ds1a.htm

[27] Amgen. (2012, December 10). Amgen to acquire deCODE Genetics, a global leader in human genetics. https://www.amgen.com/newsroom/press-releases/2012/12/amgen-to-acquire-decode-genetics-a-global-leader-in-human-genetics

[28] King EA, Davis JW, & Degner JF (2019). Are drug targets with genetic support twice as likely to be approved? Revised estimates of the impact of genetic support for drug mechanisms on the probability of drug approval. PLoS Genetics, 15(12), e1008489. 10.1371/journal.pgen.1008489 PMC6907751 31830040

[29] Amgen. (2022, December). Targeting obesity using biology, not behavior. https://www.amgen.com/stories/2022/12/targeting-obesity-using-biology-not-behavior;

[30] Kurki MI, Karjalainen J, Palta P, Sipilä TP, Kristiansson K, Donner KM, Reeve MP, Laivuori H, Aavikko M, Kaunisto MA, Loukola A, Lahtela E, Mattsson H, Laiho P, Della Briotta Parolo P, Lehisto AA, Kanai M, Mars N, … Palotie A (2023). FinnGen provides genetic insights from a well‐phenotyped isolated population. Nature, 613(7944), 508–518. 10.1038/s41586-022-05473-8. 36653562 PMC9849126

[31] Lehtimäki H, Helén I, Snell K, Eriksson P, & Montnen T (2017). Commercialization of biobank data: The case Auria Biobank. In Sardana GD & Thatchenkery T (Eds.), Organization development through strategic management (pp. 9–18). https://www.researchgate.net/publication/312628647_Commercialization_of_biobank_data_The_case_of_Auria_Biobank

[32] Tempus. (2017, July 21). Tempus and Rush University Medical Center partnering to expand hospital’s first biobank. https://www.tempus.com/news/pr/tempus-and-rush-university-medical-center-partnering-to-expand-hospitals-first-biobank/

[33] Rush University Medical Center. (2018, October 23). Rush collaborates with tech firm Tempus to expand cancer biorepository. https://www.rush.edu/news/rush-collaborates-tech-firm-tempus-expand-cancer-biorepository/

[34] All of Us Research Program. Controlled tier data access training materials. Accessed October 2, 2024.

[35] Lee YS, Lord G, Szatrowski A, Maggio ZA, Knutson AK, Hedges JR, & Khomtchouk BB (2024). The Molecular‐Social‐Genetic Determinants of Cardiovascular Health in Pacific Islanders. JACC Journals, 4(7) 559–565. https://www.jacc.org/doi/10.1016/j.jacasi.2024.04.012 10.1016/j.jacasi.2024.04.012 PMC11291382 39101109

[36] Marie McSorley AM, Wheatley A, & Pagán JA (2023). A call to increase health data availability in US territories—Not too small to count. JAMA Health Forum, 4(9), e233088. 10.1001/jamahealthforum.2023.3088 37738063

[37] Our Health Study. (2024). Frequently asked questions. Retrieved May 29, 2024, from https://ourhealthstudy.org/faq

