# Supplementary material for: Ethical considerations for biobanks serving underrepresented populations
Source: Bioethics. Author manuscript; Available in PMC 2026 Mar 1. (PMC11831713; doi:10.1111/bioe.13381)
Supplement: Supinfo [file NIHMS2039121-supplement-Supinfo.docx]

**Supplemental Material**

Supplemental Table 1: Potential types of data that can be extracted from different tissue types often housed in biobanks. List is not exhaustive.

| **Tissue type** | **Data that can be potentially extracted** |
| --- | --- |
| Whole Blood | Complete blood count (CBC), differential count (DC), cholesterol levels, glucose levels, immune markers, genomic DNA, RNA |
| Plasma | Proteins (e.g., albumin, globulin), hormone levels, electrolytes, cytokines, metabolites, viral RNA/DNA, antibodies |
| Serum | Hormone levels, enzyme activities, cholesterol, triglycerides, disease biomarkers, antibodies |
| Urine | Kidney function markers (e.g., creatinine, urea), electrolyte levels, proteinuria, glucose, drug metabolites, infection markers (e.g., leukocytes, nitrites) |
| Saliva | Cortisol levels, genomic DNA, microbiome analysis, hormone levels, proteins, antibodies (e.g., IgA), infection markers |
| Tissue Biopsy | Genomic DNA, RNA expression profiles, proteomics, histopathological data, tumor markers, epigenetic data |
| Cerebrospinal Fluid | Biomarkers for neurodegenerative diseases (e.g., amyloid-beta, tau proteins), infection markers, immune cell profiles, metabolites |
| Hair | Hormonal analysis (e.g., cortisol), drug metabolites, genomic DNA, biomarkers for chronic stress |
| Nail Clippings | Trace elements (e.g., zinc, arsenic), long-term drug exposure, biomarkers for environmental exposure |
| Feces | Microbiome composition, metabolites, gut inflammation markers, digestive enzyme activity |
| Sweat | Electrolyte analysis (e.g., sodium, chloride), drug metabolites, biomarkers for stress or hydration |
| Breast Milk | Immune factors (e.g., antibodies, cytokines), microbiome analysis, nutrients (e.g., lactose, lipids), hormone levels |
| Amniotic Fluid | Fetal DNA for genetic testing, proteomics, metabolic markers, infection markers |
| Buccal Swab | Genomic DNA, epigenetic modifications, microbiome analysis |
| Semen | Sperm count and motility, genomic DNA, hormone levels, infection markers |
| Bone Marrow | Hematopoietic stem cells, genetic mutations, biopsies used for diagnostic purposes, immune cell profiles |
